# Long Non-Coding RNAs: Potential Biomarkers and Targets for Hepatocellular Carcinoma Therapy and Diagnosis

**DOI:** 10.7150/ijbs.50730

**Published:** 2021-01-01

**Authors:** Donghong Yuan, Yu Chen, Xiaobing Li, Jing Li, Yueshui Zhao, Jing Shen, Fukuan Du, Parham Jabbarzadeh Kaboli, Mingxing Li, Xu Wu, Huijiao Ji, Chi Hin Cho, Qinglian Wen, Wanping Li, Zhangang Xiao, Bo Chen

**Affiliations:** 1 Laboratory of Molecular Pharmacology, Department of Pharmacology, School of Pharmacy, Southwest Medical University, Luzhou, Sichuan, China.; 2 South Sichuan Institute of Translational Medicine, Luzhou, Sichuan, China.; 3 Department of Oncology, Hospital of Chengdu University of Traditional Chinese Medicine, Chengdu, Sichuan, China.; 4 Department of Oncology and Hematology, Hospital (T.C.M) Affiliated to Southwest Medical University, Luzhou, Sichuan, China.; 5 Department of Oncology, The Affiliated Hospital of Southwest Medical University, Luzhou, Sichuan, China.; 6 Science and Technology Achievement Incubation Center, Kunming Medical University, Kunming, Yunnan, China.

**Keywords:** LncRNA, HCC, biomarkers, therapeutic and diagnostic targets

## Abstract

Hepatocellular carcinoma (HCC) is one of the leading causes of cancer-related death worldwide. Increasing studies showed that long non-coding RNAs (lncRNAs), a novel class of RNAs that are greater than 200 nucleotides in length but lack the ability to encode proteins, exert crucial roles in the occurrence and progression of HCC. LncRNAs promote the proliferation, migration, invasion, autophagy, and apoptosis of tumor cells by regulating downstream target gene expression and cancer-related signaling pathways. Meanwhile, lncRNA can be used as biomarkers to predict the efficacy of HCC treatment strategies, such as surgery, radiotherapy, chemotherapy, and immunotherapy, and as a potential individualized tool for HCC diagnosis and treatment. In this review, we overview up-to-date findings on lncRNAs as potential biomarkers for HCC surgery, radiotherapy, chemotherapy resistance, target therapy, and immunotherapy, and discuss the potential clinical application of lncRNA as tools for HCC diagnosis and treatment.

## Introduction

Hepatocellular carcinoma (HCC), one of the primary liver cancers, ranks sixth in the incidence of global malignant tumors and ranks third in the mortality rate 1. The occurrence of HCC is a complicated process caused by several factors. The main factors include chronic hepatitis viruses, and chronic hepatitis caused by heavy drinking, diabetes, and nonalcoholic fatty liver disease 2. The treatment strategies for HCC proposed by clinical guidelines include tumor resection and liver transplantation by surgery, transhepatic arterial chemotherapy and embolization (TACE), and radiotherapy. Recent decades, new strategies, such as molecular targeting treatment, and immunotherapy, were applied for HCC treatment worldwide 3-5. However, the impact of such improvement on population mortality from HCC is limited, since the five-year survival is still low 6. Hence, early diagnosis, early treatment, and more effective treatment for HCC are particularly important.

**Figure 1:**
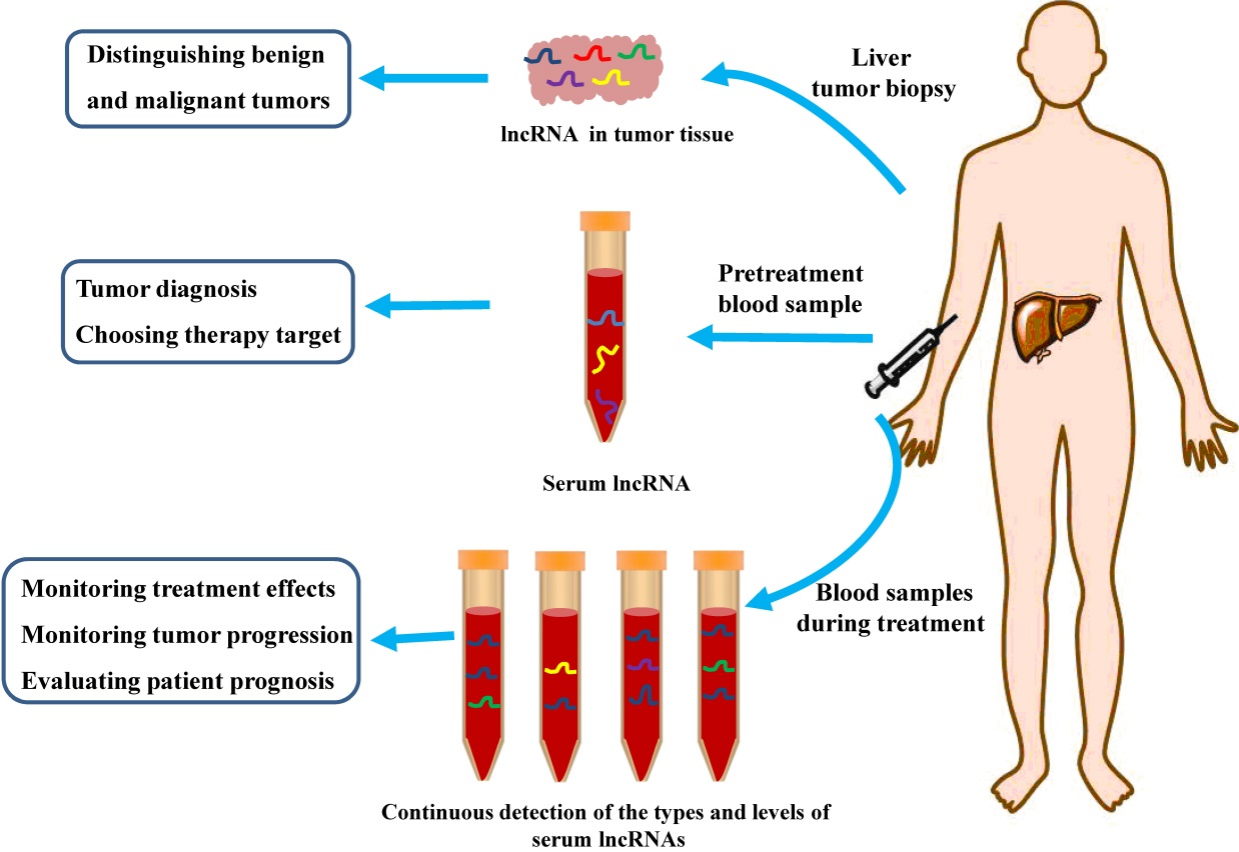
** Potential Clinical Applications of LncRNAs as Biomarkers for Diagnosis and Treatment of HCC.** LncRNAs can be used as an indicator to distinguish benign and malignant tumors. Serum lncRNAs can be used as potential biomarkers for HCC diagnosis, or be used as one of the reference conditions for clinicians to choose suitable therapeutic strategy. Continuous collection of blood samples and check the types and levels of serum lncRNA during treatment can be an important indicator of the effectiveness of treatment, as well as monitoring disease progression and predicting the prognosis of HCC patients.

Long non-coding RNAs (lncRNAs) are a class of RNAs with length longer than 200 nucleotides and lacking the ability to encode proteins 7. Previous studies have suggested that non-coding RNAs are “transcriptional noise” production during gene expression, however, with the deepening of studies, researchers found that the proportion of non-coding genes in human genome is far more higher than the proportion of protein-coding genes in human genome, which only accounts for less than 2% 8. In recent years, with the development of high-throughput sequencing technologies, lncRNAs have been revealed to be abnormally expressed in tumor tissues. The abnormal expression of lncRNA is closely concerned with cancer occurrence, progression, and prognosis, and studies on lncRNAs brings new ideas for the study of the pathogenesis of tumor and for the exploration of new diagnostic and therapeutic methods **(Figure 1)** 9, 10. More and more studies showed that lncRNAs, as oncogenes (**Table 1**) 11-56 or suppressors (**Table 2**) 57-76, play crucial role in regulating the progression, metastasis, and invasion of HCC.

In this review, we overview and discuss the up-to-date findings of the role of lncRNAs as potential diagnostic biomarkers and therapeutic targets for HCC diagnosis and treatment.

## Serum LncRNAs as Potential Diagnostic Biomarkers for HCC

Serum alpha fetal protein (AFP) is the common and golden standard tumor biomarker for screening and diagnosing of HCC, however, recent studies showed that the sensitivity and specificity of using AFP as biomarker for early diagnosis of HCC are not satisfactory 77. Novel biomarkers need to be searched for improving the early diagnosing rate of HCC. More and more studies showed that serum lncRNAs, such as ENSG00000258332.1, LINC00635, small nucleolar RNA host gene (SNHG)1, lncRNA uc007biz.1 (LRB1), highly up-regulated in liver cancer (HULC), linc00152, and urothelial cancer associated (UCA) 1 , are potential biomarkers for early diagnosis of HCC, meanwhile, combined detection of the serum levels of these lncRNAs with serum levels of AFP showed highest sensitivity and accuracy for early diagnosis of HCC 78-82.

Serum levels of several lncRNAs were upregulated in HCC patients. Xu et al. detected serum exosomes levels of LINC00635 and ENSG00000258332.1 in HCC patients, hepatic cirrhosis patients, chronic hepatitis B patients, and healthy subjects, and found that these lncRNAs were up-regulated in HCC group, and high serum levels of these lncRNAs are associated with poor prognosis 78. Moreover, the combined detection of serum levels of LINC00635, ENSG00000258332.1, and l AFP showed a high sensitivity for the diagnosis of HCC 78. Gao et al. found that the plasma levels of lncRNA SNHG1 was higher in HCC patients 79. Further investigation demonstrated that combined detection of SNHG1 and AFP levels improves the accuracy of the early diagnosis of HCC 79. Similar study by Li et al. found that serum levels of HULC and Linc00152 were higher in HCC patients, and combined detection of serum levels of HULC, Linc00152, and AFP improves the diagnostic accuracy of HCC 80.

The upregulation of some lncRNAs also can be as potential biomarkers for diagnosing of HCC tumor stages and overall survival. Wang et al. found that the serum levels of LRB1, accompanied by the levels of protein markers AFP, were obviously increased in HCC patients, and using LRB1 combined with AFP as biomarkers can significantly improve the diagnostic accuracy of HCC 81. Further investigation found that the serum LRB1 levels were positively correlated with HCC tumor stages, and were negatively associated with overall survival 81. Zheng et al. found that UCA1, the serum levels of which was upregulated in HCC patient, is a good candidate marker for HCC diagnosis, and high serum UCA1 levels were correlated with advanced tumor, node, and metastasis (TNM) stage, high tumor grade, and high tumor size 82.

## LncRNAs as Predictors for Recurrence and Survival Time after Surgical Treatment of HCC

Surgical treatments, including liver transplantation (LT) and partial liver resection (LR), are efficient methods for HCC treatment; however, the post-treatment prognosis remains poor due to metastasis and recurrence. Recent studies showed that lncRNAs have a great effect on the prediction of recurrence and survival time in HCC patients after surgery.

### HOXA Transcript at the Distal Tip (HOTTIP)

Wu et al. collected blood and tumor tissue samples from 155 HCC patients who underwent LT treatment, the authors found that HOTTIP have a high expression level in HCC tissues 83. COX multivariate analysis showed that high HOTTIP expression was independent risk factor for tumor recurrence after LT and was associated with shorter overall survival time. Further *in vitro* studies indicated that down-regulateded HOTTIP expression impairs the invasiveness and increases the chemosensitivity of tumor cells. In addition, two single nucleotide polymorphisms (SNPs) (rs2071265 and rs142077238) in the HOTTIP gene were genotyped in 102 peripheral blood samples of HCC patients, COX regression analysis found SNP rs2071265 is an independent predictor for HCC recurrence and is associated with early recurrence of HCC patients. These results indicate that HOTTIP expression levels and SNP type are predictive indicators for tumor recurrence after liver transplantation in HCC patients 83.

### lncRNA Associated with Microvascular Invasion in Hepatocellular Carcinoma (MVIH)

Yuan et al. found that the MVIH expression levels were markedly upregulated in cancer tissues of HCC patients undergoing hepatectomy 84. Further analyses demonstrated that patients with higher MVIH expression levels showed worse relapse-free survival and overall survival, and higher MVIH expression level was a risk factor for early postoperative recurrence in HCC patients 84.

### Nuclear Paraspeckle Assembly Transcript 1 (NEAT1)

NEAT1 is oncogenic lncRNA in diverse kinds of tumorigenesis, including gastric cancer, breast cancer, lung cancer and colorectal cancer 85. Recent studies showed that NEAT1 can be used as an indicator of outcome evaluation in patients with HCC after tumor resection. Zhao et al. examined the expression level of NEAT1 in the liver cancer tissues of 86 primary liver cancer patients undergoing liver cancer resection, they found that NEAT1 expression was up-regulated in liver cancer tissues, and silencing NEAT1 arrested HCC cell proliferation, NEAT1 expression level is correlated with overall survival rate, microvessel formation-related invasion (MVI) and TNM stage 86.

### Metastasis Associated Lung Adenocarcinoma Transcript 1 (MALAT1)

MALAT1 is the most widely researched lncRNAs in the treatment of cancer 87. Studies have shown that the higher expression of MALAT1 is closely associated with the progression and prognosis of patients with HCC. Lai et al. found that MALAT1 was upregulated in HCC tissues of patients undergoing liver transplantation, in comparison to the matched adjacent tissues. Further studies demonstrated that patients with high MALAT1 expression showed significantly lower recurrence survival rate than patients with low MALAT1 expression. In addition, multiple regression analysis showed that the expression level of MALAT1 could be used as a new independent predictor of recurrence-free survival after liver transplantation in patients with HCC 88.

### HOX Transcript Antisense RNA (HOTAIR)

HOTAIR is the antisense strand of HOX gene cluster, and is an oncogenic lncRNA in cancer 89. Yang et al. found that tumor tissues of HCC patients with LT showed high expression levels of HOTAIR 90. Survival analyses revealed that 3-year cumulative recurrence-free survival rate of HCC patients with high expression of HOTAIR was enormously reduced, in addition, high levels of HOTAIR was also susceptible to early recurrence in patients with HCC undergoing surgical resection 90.

## LncRNAs as Radiotherapy Biomarkers in HCC

**Figure 2:**
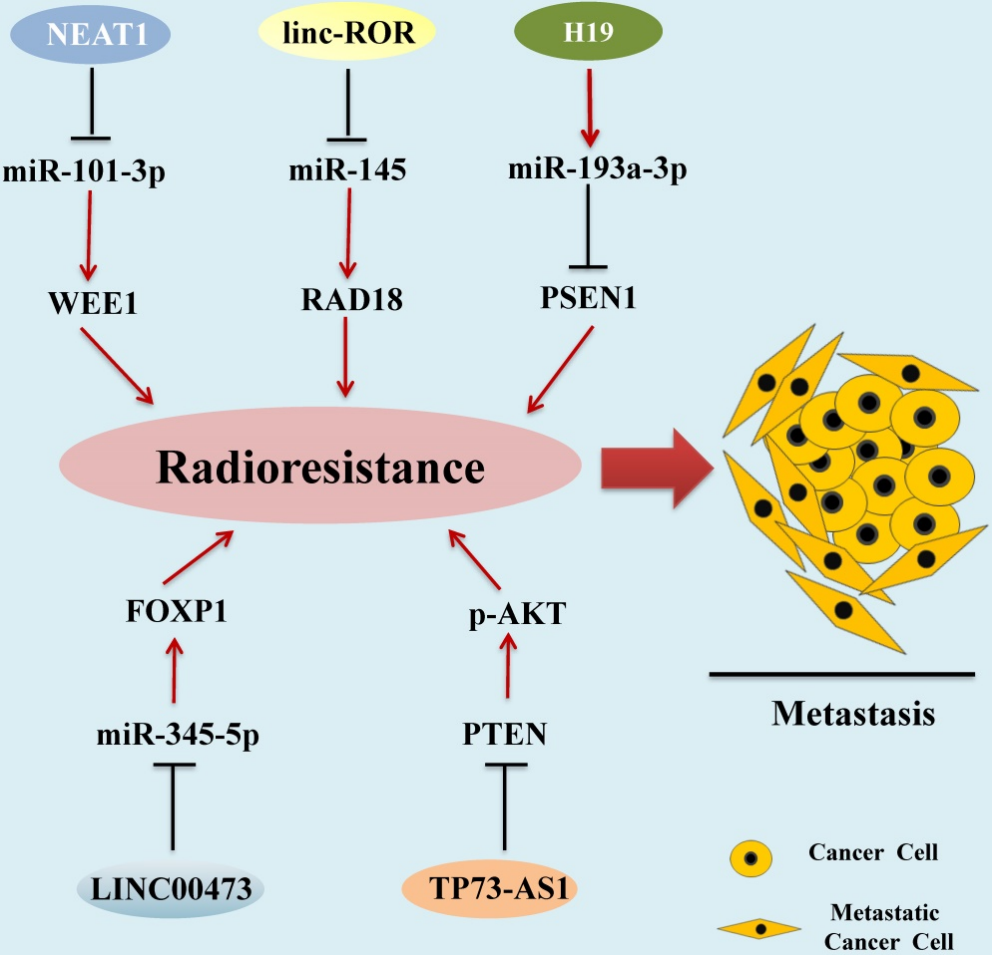
** LncRNAs as Radiotherapy Biomarkers in HCC.** Aberrant expression of lncRNAs associated with the development of HCC radioresistance, leading to metastasis and progression of HCC. lncRNAs mediate functions by regulating miRNA expression via diverse molecular mechanisms.

Radiotherapy is one of the main traditional methods for HCC treatment 91. Radiotherapy-resistant has become a main clinical problem for the successful treatment of HCC 92. Recent studies demonstrated that aberrant expression of some kinds of lncRNAs are correlated with the development of HCC radioresistance (**Figure 2**).

### Nuclear Paraspeckle Assembly Transcript1_2 (NEAT 1_2)

In HCC tissues and cells, Chen et al. found that the levels of lncRNA NEAT1_2 and oncogenic protein WEE1 was upregulated 93. Knockdown of the expression levels of WEE1 and NEAT1_2 induced HCC cell apoptosis and enhances radiosensitivity. In addition, miR-101-3p was directly targeted to WEE1 and inhibited the WEE1 expression levels in HCC cells, while NEAT1_2 up-regulated the expression levels of WEE1 via sponge miR-101-3p. These studies indicate that NEAT1_2/miR-101-3p/WEE1 axis is a potential target signaling pathway for enhancing the sensitivity of HCC radiotherapy 93.

### Long Noncoding RNA Regulator of Reprogramming (Linc-ROR)

Chen et al. investigated the roles of the linc-ROR in HCC, and found that linc-ROR promotes HCC metastasis by mediating epithelial-mesenchymal transition. Further study found that radiation-tolerant HCC cells showed higher levels of linc-ROR and lower levels of γ-H2AX, a DNA damage marker 94. In addition, luciferase reporter assay showed that linc-ROR acts as a molecular sponge of miR-145 in HCC cells, and overexpression of linc-ROR can inhibit miR-145 expression and reduce translational inhibition of downstream miR-145 target RAD18, which is an important DNA repair factor, and radioresistance mediator 95. Linc-ROR modulates RAD18 expression by competitively sponging miR-145, thereby inducing radioresistance of HCC cells 96. Therefore, Linc-ROR can be used as a biomarker and as a potential target for HCC radiotherapy.

### SUMO1 Pseudogene 3 (SUMO1P3)

SUMO1P3 is a novel identified lncRNA that was up-regulated in several types of cancer, including gastric cancer, bladder cancer, and non-small cell lung cancer, and is a prognostic and therapeutic target these cancers 97-99. Zhou et al. explored the effect of SUMO1P3 in HCC, and discovered that the expression of SUMO1P3 was markedly increased in HCC tissues and cells 100. *In vitro*, knockdown of SUMO1P3 expression of HCC cells, and explosion of these cells to radiation could significantly reduce the survival rates of HCC cells, indicating that knockdown of SUMO1P3 promotes the radiosensitivity of HCC cells. Meanwhile, knockdown of SUMO1P3 could also inhibit the growth of tumor cells, reduce invasiveness and promote apoptosis 100.

### H19 Imprinted Maternally Expressed Transcript (H19)

LncRNA H19 was firstly identified as mammalian development regulator 101, and is an oncogenic lncRNA in cancers, including HCC 102. Ma et al. found that the H19 expression level in radiation-sensitive HCC cells was obviously lower than that in radiation-resistant HCC cells 103. Suppression of lncRNA H19 expression promotes radiation induced HCC cell apoptosis. Mechanistic studies demonstrated that lncRNA H19 directly targeted miR-193a-3p to suppress radiation induced HCC apoptosis 103. Furthermore, PSEN1 can be directly targeted by miR-193a-3p, low expression of PSEN1 was negatively related to radiosensitivity of HCC cells, so the H19/miR-193a-3p/ PSEN1 axis can be used as a potential pathway to ameliorate the curative effect of radio‐therapies for HCC 103.

### LINC00473

Zhang and colleagues detected the expression level of LINC00473 in HCC cell lines and normal hepatocytes, and the results indicated that the expression level of LINC00473 in HCC cell lines was significantly higher than that of normal hepatocytes 104. Subsequently, SK-HEP-1 and Huh-7 cells were exposed to a gradient dose of radiation for 6 hours and then measured the level of LINC00473 again. The results showed that when the radiation dose reached 6 Gy, LINC00473 expression level was proportional to the radiation time. Further experiments found that knocking down LINC00473 inhibited the proliferation of HCC cells, induced apoptosis, and increased its radiosensitivity 104. In terms of mechanism research, it was discovered that LINC00473 targets and inhibits the expression of miR-345-5p, and the overexpression of miR-345-5p can enhance the radiosensitivity of HCC cells 104. Subsequent studies demonstrated that miR-345-5p negatively regulates forkhead box P1 (FOXP1), and silencing the FOXP1 gene hindered HCC radioresistance. These data indicate that the LINC00473/miR-345-5p/FOXP1 axis regulates HCC cell radioresistance 104.

### LncRNA P73 Antisense RNA 1T (lncRNA TP73-AS1)

LncRNA TP73-AS1 is located on human chromosome 1p36 105, many evidences have identified lncRNA TP73-AS1 as an oncogene of liver cells 106, 107. Song and colleagues found that TP73-AS1 expression was significantly increased in HCC cell lines and tissues 108. *In vitro* studies showed that lncRNA TP73-AS1 down-regulated the expression of PTEN, enhanced the phosphorylation level of AKT, and reduced the radiosensitivity of HCC cells. The same results have also appeared *in vivo* experiments. After silencing lncRNA TP73-AS1, the tumor growth rate of mice was significantly slowed down, and the tumor volume was also significantly smaller than the control wildtype group 108.

## LncRNAs and Chemotherapy Resistance in HCC

**Figure 3:**
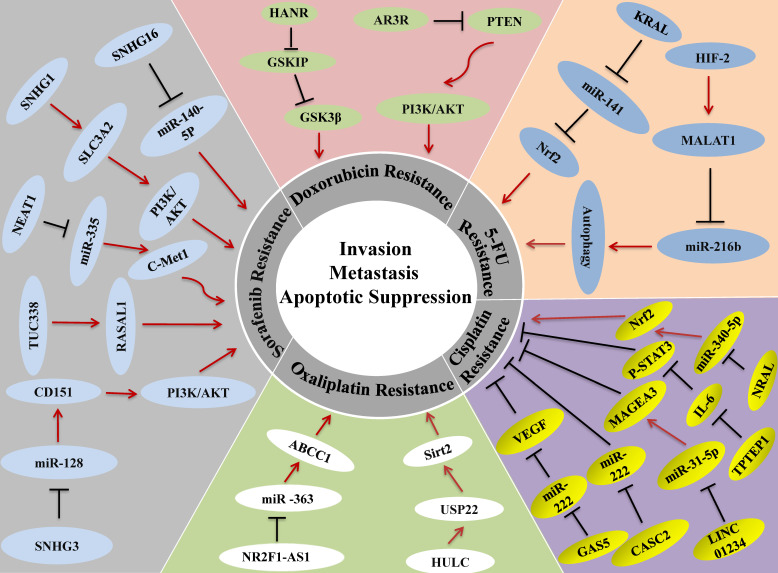
** lncRNAs in Drug Resistance of HCC.** Up- or down-regulation of LncRNA could enhance the resistance of HCC cells to various chemotherapeutic and targeted drugs, leading to tumor cell invasion, metastasis or the inhibition of cancer cell apoptosis.

Chemotherapy is a traditional and efficient method for cancer therapy and the most common drugs for treating HCC include 5-fluorouracil (FU), cisplatin (CDDP), oxaliplatin, doxorubicin, gemcitabine, capecitabine, and mitoxantrone 109. Although single or a combination of chemo drugs has proper effects on HCC, overtime use of chemotherapy for cancers can develop resistance to chemo-drugs, which greatly limits the efficacy of chemotherapy 110. The molecular mechanism of chemotherapy resistance is related to apoptosis evasion, stem cell activation, enhanced DNA repair, and topoisomerase activation; these changes can promote drug inhibition, drug degradation, or drug target alteration, and finally help cancer cells to evade the effects of chemotherapy 111. More and more investigations demonstrated that lncRNAs are new targets for the treatment of HCC drug resistance. In this part, we outline how lncRNAs participate in drug resistance of 5-FU, cisplatin, oxaliplatin, and doxorubicin in HCC (**Figure 3**).

### 5-FU Resistant-Related LncRNAs

#### Keap1 Regulation-Associated LncRNA (KRAL)

By performing lncRNA microarray analyses, Wu et al. found that the mRNA levels of KRAL were significantly reduced in 5-FU-resistant HCC cells 112. Subsequent studies showed that KRAL inhibits Keap1 mRNA degradation by binding with the 3'-untranslated region (UTR) of Keap1 113. Keap1 is a potential mediator for drug sensitivity in cancer therapy by suppressing the activity of Nrf2 114. Previous studies have shown that miR-141 promotes Keap1 mRNA degradation 115, and the levels of miR-141 were significantly increased improved in SMMC-7721/5-FU and HepG2/5-FU cells5-FU-resistant HCC cells 113. Moreover, miR-141 downregulates the expression of Keap1 by activating Nrf2-dependent antioxidant pathway to confer HCC cell resistance to 5-FU 113. Therefore, the KRAL/miR-141/Keap1/Nrf2 axis pathway has potential great application prospects as a therapeutic target for overcoming HCC cells 5-FU resistance in HCC cells.

#### Metastasis Associated Lung Adenocarcinoma Transcript 1 (MALAT1)

LncRNA MALAT1 was initially identified as an indicator index of poor clinical outcome in early-stage non-small cell lung cancer (NSCLC) patients 116. As the study progressed, it was found that MALAT1 not only be useful for lung cancer but also has an essential effect on other human cancers, including HCC 117. MALAT1 regulates tumor progression and metastasis by providing competitive endogenous RNA, interacting with polycomb repressive complex2 (PRC2) complexes, binding and inactivating TEAD, and regulating multiple signaling pathways 87, 118, also associated with cancer resistance 119, 120. MALAT1 can be induced in tumor cells under hypoxic conditions 121, Yuan *et al.* first compared the expression of MALAT1 in 5-FU-sensitive and resistant HCC cell lines, and the results revealed that MALAT1 mRNA levels and the levels of hypoxia-inducible factor (HIF)-2α were significantly increased in 5-FU resistant cells 16. Meanwhile, knockdown of HIF-2α in HCC cells abolished hypoxia-induced upregulation of MALAT1; these results indicate that HIF-2α promotes upregulation of MALAT1 in HCC cells 16. Previous studies reported that enhanced autophagy is one of the chemical metabolism mechanisms of HCC cells 122, 123, and subsequent by performing bioinformatics analysis revealed that miR-216b is a potential MALAT1 target 16. Further studies have shown that MALAT1 promotes autophagy by inhibiting the expression of miR-216b and enhances the 5-FU chemical metabolism in HCC cells 16.

### Cisplatin (CDDP) Resistant-Related LncRNA

#### Nrf2 Regulation-Associated LncRNA (NRAL)

Wu et al. established the drug-resistant HCC cell lines and performed lncRNA microarray analysis to explore the expression patterns of lncRNAs in CDDP-resistant cancer cells 124. Data showed that levels of NRAL in CDDP-resistant HCC cells was greatly upregulated 124. Moreover, NRAL could bind with mir-340-5p, a tumor suppressor miRNA 125, to downregulate its expression. Further investigation showed that NRAL unites with miR-340-5p through endogenous "sponge", up-regulates its target Nrf2 expression, activates Nrf2-dependent antioxidant pathway leading to CDDP resistance in HCC cells 124. The NRAL/miR-340-5p/Nrf2 axis may supply novel ideas for overcoming resistant to CDDP in HCC cells.

#### Growth Arrest Specific 5 and Cancer Susceptibility 2 (GAS5 and CASC2)

GAS5 was initially identified in a subtractive complementary DNA (cDNA) library, which was used to sequence RNA expressed in growth-arrested cells 126. Subsequently, more and more literatures show that GAS5 plays a vital role in the progress of HCC 61-63. Zhao et al. found that the expression of GAS5 in CDDP-resistant HCC patients and cell lines was significantly reduced, and the survival rate of HCC patients with low GAS5 expression was significantly decreased. Subsequent experimental results showed that overexpression of GAS5 increased the sensitivity of HepG2/CDDP and Huh7/CDDP cells to CDDP. In the mechanism study, it was found that GAS5 inhibited the expression of miR-222 gene, inactivating the VEGF signaling pathway and making CDDP-resistant HCC cells sensitive to CDDP 127. In a recent study, it was found that another lncRNA CASC2 can also enhance the sensitivity of CDDP-resistant HCC cells to CDDP by inhibiting the expression of miR-222 gene 59.

#### LINC01234

LINC01234 has been proven to be an indicator of prognosis in ovarian cancer 128. Yunhao Chen et al. analyzed the gene expression profiles of 374 HCC and 50 normal livers in the Cancer Genome Atlas database. They found that MAGEA3 and LINC01234 are both highly expressed in HCC tissues. Further analysis showed that MAGEA3 may be a downstream target of miR-31-5p. Next experiments confirmed that the down-regulation of MAGEA3 inhibits the proliferation and migration of HCC cells, induces apoptosis, and weakens the chemical resistance of HCC cells to cisplatin. Bioinformatics analysis suggests that LINC01234 can bind to miR-31-5p, further experiments found that miR-31-5p depletion can reverse the antitumor and chemosensitization caused by LINC01234 silence. These experiments confirmed that the reduction of LINC01234 up-regulated miR-31-5p, inhibiting the expression of MAGEA3, thereby increasing the chemical sensitivity of HCC cells to cisplatin 129.

#### TPTE Pseudogene 1 (TPTEP1)

TPTEP1 is located on chromosome 22 and is regulated by DNA methylation in cancer 130. Ding and colleagues used RNA-seq to analyze the differential expression of lncRNA in QGY7703 cells treated with cisplatin, and determined that lncRNA TPTEP1 had the highest expression level after cisplatin treatment 131. Subsequent functional gain and loss experiments in liver cancer cell lines showed that the up-regulation of TPTEP1 inhibited the proliferation and invasion of HCC cells and enhanced the apoptosis induced by cisplatin, the opposite result appeared after silence TPTEP1 131. Further analyses found that TPTEP1 inhibits IL-6-induced phosphorylation of STAT3, thereby inhibiting the transcriptional activity of STAT3 and increasing the sensitivity of liver cancer cells to cisplatin. This result was also verified in *in vivo* experiments, where TPTEP1 inhibited tumor growth in mice, reduced the number of lung and liver metastases, and was down-regulated in mouse tumor tissues 131.

### Oxaliplatin Resistant-Related LncRNAs

#### NR2F1 Antisense RNA 1 (NR2F1-AS1)

Huang et al. performed microarray analysis on four pairs of oxaliplatin-resistant and sensitive HCC cells, and discovered that NR2F1-AS1 was highly expressed in oxaliplatin-resistant HCC cells 132. Moreover, NR2F1-AS1 was also highly expressed in oxaliplatin-resistant HCC tissues 132. Knockdown NR2F1-AS1 inhibits HCC cell resistance to oxaliplatin, leading to suppress the migration, invasion and tumor growth of HCC cells 132. NR2F1-AS1 mechanically induced HCC cell resistance to oxaliplatin by downregulating the expression of miR-363, which further induced high expression of multidrug resistance-associated protein 1 132.

#### Highly Up-regulated in Liver Cancer (HULC)

HULC was first identified as the most up-regulated lncRNA in HCC 133. Xiong et al. compared the levels of HULC in HCC tissues and corresponding adjacent non-cancerous liver tissues, and found that HULC mRNA and protein levels were significantly up-regulated in HCC tissues 134. Increasingly evidence showed that HULC has a non-negligible role in the growth, invasion, migration, prognosis and chemoresistance of HCC 135. Using a nude mouse xenograft model, investigators found that HULC knockdown significantly increased the sensitivity of oxaliplatin chemotherapy, which may mediate by the stabilization of stress response gene sirtuin1 (sirt1) 134. Sirt1 plays important roles in drug resistance by decreasing drug penetration 136, so the HULC-Sirt1 axis is a new target for overcoming resistance of HCC cells to chemotherapy drug oxaliplatin.

### Doxorubicin Resistant-Related LncRNAs

#### HCC Associated Long Non-Coding RNA (HANR)

Xiao et al. found a novel lncRNA named as HCC associated long non-coding RNA (HANR), and found that the expression levels of HANR in HCC cell lines and HCC tissues were up-regulated. In addition, Kaplan-Meier analysis showed that high HANR expression was connected with poor prognosis 137. Further studies showed that HCC cell resistance to doxorubicin is positively regulated by HANR 137. HANR promotes HCC cells resistance to doxorubicin by regulating the activity of GSK3β 137, which plays important roles in drug-induced cancer cell apoptosis 138. These findings indicate that HANR is a promising therapeutic target for HCC cell resistant to doxorubicin.

#### LncRNA Regulator of AKT Signaling Associated with HCC and RCC (LncARSR)

LncARSR was firstly found in renal cell carcinoma (RCC) cells, and was related with resistance to sunitinib in RCC patients 139. Li et al. found that lncARSR was significantly up-regulated in HCC tissues 140. Further studies showed that overexpression of lncARSR significantly reduced the sensitivity of HCC cells to doxorubicin 140. Moreover, knockdown of lncARSR rescues the sensitivity of HCC cells to doxorubicin treatment 140. PTEN is a well-known tumor suppressor by inhibiting the activation of the PI3K/Akt pathway, and is associated with doxorubicin resistance in mutiple cancers, including liver cancer 141. Bioinformatics analysis and RNA pull-down experiment found that PTEN is a downstream target of lncARSR, and lncARSR negatively regulates PTEN expression in HCC tissues. Further investigations showed that PI3K/Akt pathway was involved in lncARSR-related doxorubicin resistance in HCC, as inhibition of PI3K/Akt pathway reversed lncARSR overexpression-induced doxorubicin resistance 140. These results indicate that lncARSR mediates doxorubicin resistance by downregulating the PTEN expression levels and subsequently reactivating PI3K/Akt signaling pathway 140.

## LncRNAs and Targeted Therapy for HCC

Molecular targeted therapy is a new method for treating cancer that has been accepted by people in recent years. Many targeted drugs, including sorafenib (SOR), lenvatinib, regorafenib, cabozantinib, and ramucirumab, currently were used to treat HCC 142. Sorafenib, as a first-line treatment for HCC, is particularly important for patients with advanced HCC and can improve the survival time of advanced hepatocellular carcinoma patients 143. Sorafenib is an oral small-molecule multi-target tyrosine kinase inhibitor, and was approved for clinical treatment of HCC in 2007 142. Sorafenib can directly inhibit tumor proliferation, neovascularization, and promote apoptosis by inhibiting RAF/mitogen-active protein kinase/extracellular signal transduction pathways, vascular endothelial growth factor, PDGFRβ, c-kit, Flt-3 and p38 tyrosine kinase transfer, etc, 142. However, many patients still develop acquired resistance to sorafenib. Recent studies have also reported that the development of sorafenib resistance involves many different pathways 144. Emerging evidence indicates that multiple lncRNAs are involved in sorafenib resistance (**Figure 3**).

### Small Nucleolar RNA Host Gene 1 (SNHG1)

SNHG1 is highly expressed in HepG2 and Huh7 cells 145, the study found that compared with untreated Huh7 cells, the SNHG1 level in the nuclear fraction of Huh7 cells incubated with sorafenib was significantly increased, indicating that sorafenib can cause nuclear accumulation of SNHG1 145. In addition, it has been reported that SNHG1 activates the Akt signaling pathway by promoting the transcription of SLC3A2 146, and then by inhibiting the function of SNHG1, it was found that consumption of SNHG1 inhibits the activation of Akt signaling pathway and enhances the association with sorafenib resistance Apoptosis and autophagy 145, so SNHG1 may become a potential target for HCC treatment.

### Small Nucleolar RNA Host Gene 1 (SNHG16)

LncRNA SNHG16 is a novel cancer-related lncRNA and promotes tumor cell growth and metastasis in multiple cancers, including HCC cells and tissues 147. Ye et al. found that the SNHG16 expression levels in SOR resistant HepG2 cells are higher than the levels in SOR sensitive HepG2 cells 147. Further *in vitro* cell model and *in vivo* xenograft tumor model data showed that knockdown of SNHG16 enhanced SOR chemosensitivity in HCC 147. In addition, SNHG16 promotes SOR resistance in HCC by inhibiting miR-140-5p the expression levels 147, which was found to promote the progression and metastasis of cancers 148.

### Small Nucleolar RNA Host Gene 3 (SNHG3)

Zhang et al. found that the IC50 Value of SOR was significantly increased in SOR-treated SNHG3-overexpressing HCC cell, indicating that SNHG3 enhance SOR resistance 149. In addition, data from patients with recurrent HCC who underwent sorafenib treatment showed that patients with higher SNHG3 expression had shorter median survival time than those with lower expression of SNHG3 149. Moreover, miR-128 was confirmed as potential target for SNHG3, and overexpression of miR-128 can reduce the level of CD151, which is rescued by increased expression of SNHG3 in HCC cells 149. Previous studies have reported that CD151 activate PI3K/AKT signaling pathway to promote epithelial-mesenchymal transition (EMT) and SOR resistance 150, so SNHG3/miR-128/CD151 signaling can be used as a therapeutic target for HCC patients with SOR resistance.

### Transcribed Ultra-Conserved Region 338 (TUC338)

LncRNA TUC338 was characterized as an oncogene in several types of cancer, such as prostate carcinoma and lung cancer 151, 152. Ji et al. discovered that TUC338 was highly expressed in HCC cancer tissues and cell lines 153. The knockdown of TUC338 significantly restores SOR treatment response in HepG2-SOR resistant cells *in vitro* and in HepG2-SOR resistant xenografts *in vivo*. Further *in vitro* study showed that knockdown of TUC338 upregulated the expression level of RAS GTPase activating protein (RasGAP) gene RASAL1 153, which inhibits tumor progression by catalyzing RAS inactivation to negatively regulate the RAS signaling pathway 154.

### Nuclear Paraspeckle Assembly Transcript 1 (NEAT1)

Chen et al. found that knockdown of NEAT1 promoted SOR-induced cancer cell death 155. Bioinformatics analysis showed that NEAT1 can directly target miR-335, which suppresses the proliferation, migration of cancer cell 156, and subsequent *in vitro* experiment showed that overexpression of NEAT1 suppress the miR-335 expression in HCC cell lines 155, indicating that miR-335 is a downstream target of NEAT1. Moreover, miR-335 negatively regulates the expression of oncogene c-Met, and miR-335 inhibition or c-Met overexpression abolished NEAT1 knocking down-induced SOR sensitivity in HCC cells 155. Overall, these data demonstrated that NEAT1/miR-335/c-Met axis regulates SOR resistance in HCC cells 155. The same result was found in another study, where NEAT1 regulated the miR-204/ATG3 axis and enhanced the SOR resistance of HCC cells 157.

## LncRNAs as Key Regulators for Immunotherapy of HCC

**Figure 4:**
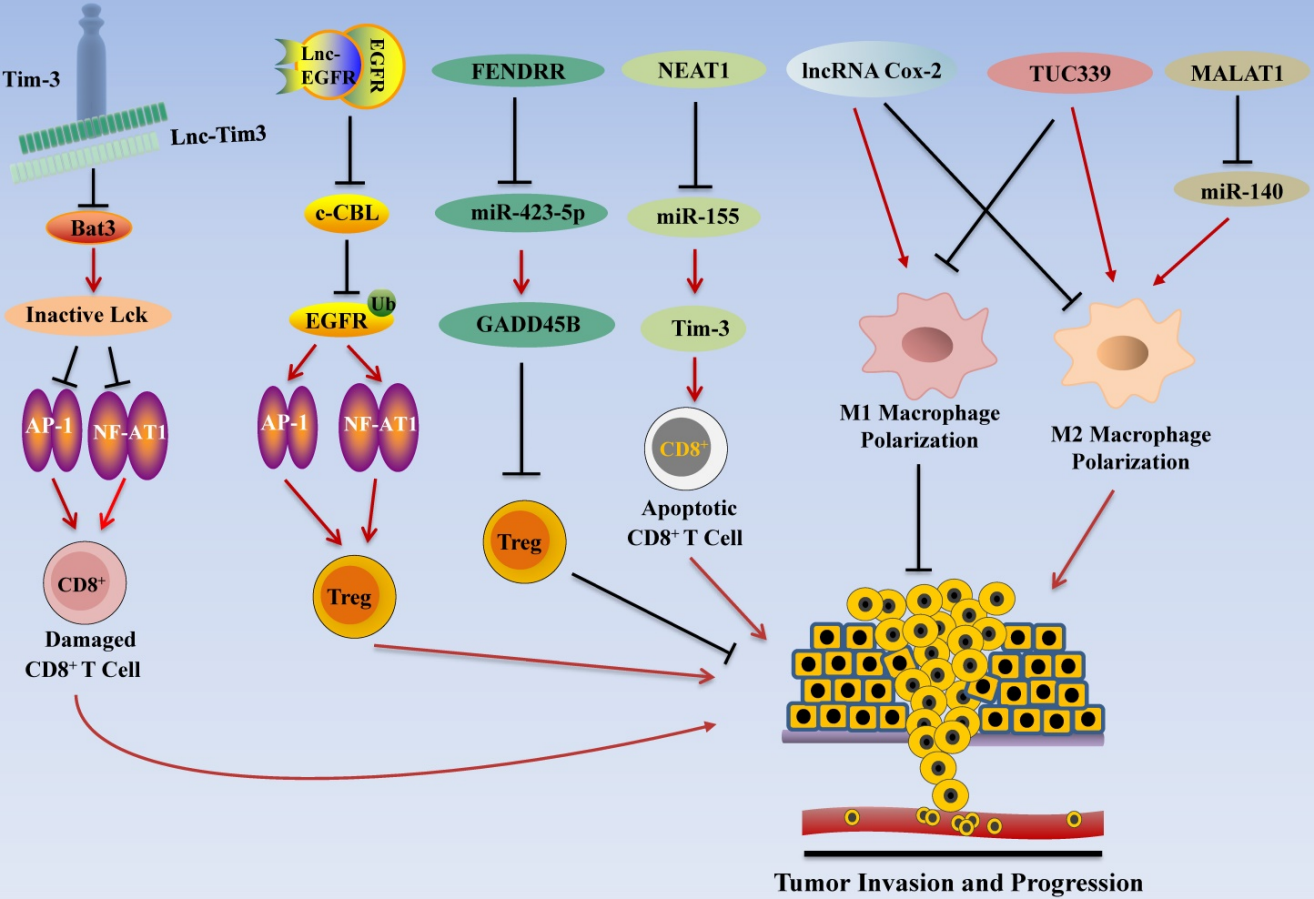
** LncRNA Regulates HCC Invasion and Progression by Modulating Tumor Immune Escape.** LncRNA promotes tumor immune escape in HCC. For example, NEAT1 inhibits the expression of miR-155 and increases the activity of Tim3, resulting in apoptosis of CD8^+^ T cells. Lnc-Tim3 inhibits NF-AT1 and AP1 signaling, thereby inducing CD8^+^ T cell failure. In addition, lnc-EGFR activates the RAS/ERK/AP1/NF-AT1 signaling pathway to promote Treg cell differentiation, thereby contributing to tumor cell immune escape.

Immunotherapy has become one of the promising options for HCC treatment following surgery, radiotherapy, chemotherapy, and targeted therapy 158. Recent studies highlighted the roles of lncRNAs in regulating immune system function in HCC by regulating T cell functions (**Figure 4**). In addition to the regulatory effect of lncRNA in T cells, it also affects macrophages and other immune cells. Tumor-associated macrophages (TAM) are important participants in tumor immunity, and the polarization of macrophages is one of the research hotspots in tumor therapy. M1 type macrophages have anti-tumor effects through direct killing mechanism and antibody-dependent cell-mediated cytotoxicity (ADCC) kills tumor cells 159. Recent studies have found that lncRNA also plays a role in the polarization process of TAM.

### LncRNA Associated with T cells in HCC

#### Nuclear Paraspeckle Assembly Transcript 1 (NEAT1)

Yan et al. found that expression levels NEAT1 were up-regulated in PBMC of HCC patients. Subsequent studies showed that knocking down of NEAT1 expression of CD8^+^ T cell suppressed cell apoptosis and enhanced the lytic activity to tumor cells by inhibiting Tim-3 expression 29. Bioinformatics analyses and further *in vitro* studies showed that miR-155 is a target of NEAT1, and miR155 inhibition alleviated knocking down of NEAT1-induced CD8^+^ T cell apoptosis, and rescued Tim-3 levels 29. These results indicate that NEAT1/miR-155/Tim-3 pathway inhibits CD8^+^ T cell function and promotes HCC immune escape, which may act as an immunotherapy target for HCC patients 29.

#### Lnc-T Cell Immunoglobulin Mucin 3 (Lnc-Tim3)

Using high-throughput screening, Ji et al. explored the associations between mRNAs and lncRNAs in the tumor infiltrated lymphocytes (TIL) of HCC patients, and found that Lnc-Tim3 was highly up-regulated and negatively correlated with the IFN-γ-secreting activity of CD8^+^ T cells in TILs 54. Further studies found that Lnc-Tim3 specific binding the intracellular domain of Tim-3 to inhibit T-cell immune response to antigen by suppressing downstream NFAT1/AP1 signaling pathway in CD8^+^ TILs, and binding of lnc-Tim3 with Tim3 leads to release of Bat3 from Tim3, thereby promoting cell cycle arrest of CD8^+^ T cells, and contributing to the survival of CD8^+^ T cells 54. These data suggest that lnc-Tim3 may affect the effectiveness of HCC immunotherapy by regulating the acquired immune system, mainly by regulating the activity of CD8^+^ T cells.

#### Lnc-Epidermal Growth Factor Receptor (Lnc-EGFR)

In order to prove the effect of lncRNAs in connecting T cells and HCC, Jiang et al. investigated the associations between mRNAs and lncRNAs in the tumor infiltrated lymphocytes (TILs) of patients with hepatocellular carcinoma and found that lnc-EGFR levels in CD4^+^ T cells were highly up-regulated 55. In addition, the expression of lnc-EGFR in the CD4^+^ T cells correlated with increased proportion of T regulatory (Treg) cells within HCC tissues 55. Further studies showed that lnc-EGFR activates RAS/ERK/AP1/NF-AT1 signaling pathway to promote Treg cell differentiation 55. It has been reported that Treg cells are related to HCC progression 160, 161; these results indicate that lnc-EGFR promotes immune escape of HCC by enhancing the differentiation of Treg cells.

#### Fetal-Lethal Non-coding Developmental Regulatory RNA (FENDRR)

Previous studies have shown that FENDRR down-regulates the expression of GPC3 and inhibits cell proliferation and migration 70. In addition, FENDRR is also considered to be one of the potential markers for HCC diagnosis 162. A recent study by Yu and colleagues found that FENDRR affects the immune escape of HCC cells. Based on microarray analysis, they discovered that the expression of FENDRR in HCC was low, and the results of qRT-PCR also showed that FENDRR was significantly lower in HCC tissues and cell lines than normal liver tissues 71. Prediction by online tools showed that miR-423-5p and GADD45B have binding sites. The difference analysis between normal samples and HCC samples revealed that FENDRR and GADD45B are positively correlated and further research found that FENDRR can interact with miR-423-5p. Subsequent treatment of Tregs co-cultured with HCC cell lines with FENDRR and miR-423-5p mimics showed that FENDR inhibited the immunosuppressive ability of Treg cells, while miR-423-5p mimics showed the opposite phenomenon. These results indicate that FENDRR competitively bind with miR-423-5p to up-regulate GADD45B, thereby inhibiting the immune escape of HCC cells mediated by Treg cell 71.

### LncRNA Associated with Macrophages in HCC

#### lncRNA Cyclooxygenase-2 (Cox-2)

LncRNA Cox-2 is a lncRNA with a size of about 50 kb 163, it is reported that Cox-2 protein is an important metabolic regulator, which plays a role in biological processes such as development, mutation, cellular immunity and cancer 164-166. The experiment of Ye et al. showed that lncRNA Cox-2 is highly expressed in M1 type macrophages, and silencing lncRNA Cox-2 inhibits the polarization of macrophages to M1 type, and enhances their ability to polarize to M2 type. Subsequently, the M1 macrophages after silencing the lncRNA Cox-2 gene were co-cultured with HCC cells, and the results suggested that the growth, invasion and migration, and immune escape capabilities of HCC cells were significantly enhanced. These results indicate that lncRNA Cox-2 inhibits the immune escape, invasion and migration of HCC cells by promoting the polarization of M1 macrophages 76.

#### TUC339

Previous studies have shown that lncRNA TUC339 is enriched in exosomes derived from HCC cells 167. Li and his colleagues extracted exosomes from PLC/PRF/5 cells and incubated them with THP-1 cells for 24 hours, after which PLC/PRF/5-derived exosomes can be internalized by THP-1 cells. Further studies showed that PLC/PRF/5 exosomes were rich in TUC339, and hepatocellular carcinoma could deliver more TUC339 to adjacent THP-1 cells 56. Subsequent studies have shown that overexpression of TUC339 promotes M2-type polarization of macrophages, and inhibit M1-type polarization of macrophages. In general, TUC339 regulates macrophage phagocytosis and M1/M2 polarization, providing insights for tumor immunotherapy 56.

#### Metastasis Associated Lung Adenocarcinoma Transcript 1 (MALAT1)

Hou and colleagues found that after knocking out MALAT1 in HCC cell lines, the ability of macrophages to polarize toward the M1 subgroup was increased, while the ability to polarize toward the M2 subgroup was weakened 168. Meanwhile, IL-6 expression level was increased while IL-10 expression level was decreased. Further study found that miR-140 is the target of MALAT1, and the expression level of miR-140 was significantly increased after MALAT1 was silenced. In addition, flow cytometry results showed that the silencing of MALAT1 increased the proportion of M1 subgroups, while miR-140 inhibitors reversed this effect 168. The same results were obtained *in vivo* experiments. The M2 macrophages polarization ability was found to be significantly weakened in mice transplanted with heterotopic liver tumors of silenced MALAT1 gene. These results suggest that MALAT1 promotes the polarization of M2 macrophages through 466 miR-140 to produce anti-cancer effect 168.

## Conclusion and Perspectives

The morbidity and mortality of HCC have been dramatically increased in recent decades. Despite advances in the diagnosis and treatment of HCC, effective treatment strategies were still not well, as metastasis and recurrence rate are still very high. More and more preclinical studies showed that lncRNAs are good diagnostic and prognostic biomarker candidates for HCC therapy. Although preclinical data showed strong relationship between lncRNAs and HCC, by now, no clinical studies were reported about using lncRNAs as biomarkers for the diagnosis and prognosis of HCC. Future clinical studies may investigate the serum lncRNA profiles to explore more specific and sensitive lncRNA as biomarkers for HCC diagnosis and prognosis. To identify new lncRNAs as biomarkers for HCC, and to elucidate the molecular mechanisms of how lncRNAs work in HCC, future studies may need to focused on the development of experimental techniques, such as new sequencing techniques, and the development of lncRNA bioinformatics databases.

While lncRNAs have great potential in the diagnosis and prognosis of HCC, we also face a series of challenges when lncRNAs are used as a potential drug candidate or therapeutic target for HCC treatment. First, the molecular weight and negative charge of nucleic acids make it difficult to make lncRNAs pass through biofilms. Second, RNA is easily degraded by RNase enzymes in the human body, recognized by the immune system, and quickly eliminated by the liver and kidneys. Finally, it is easy to lose function in the endosomes. At present, there are two main methods to solve the delivery problem: one is to modify nucleic acid molecules to stabilize and avoid recognition by the immune system, the other is to use drug delivery systems, such as protamine vector technology, nanoparticle vector technology, adeno-associated viral vector (AAV) technology, etc., among which almost all natural AAV capsid proteins can trigger effective transgene expression in the liver, so recombinant AAV targeted to the liver is a treatment HCC provides an excellent gene delivery platform. However, these delivery systems still have defects such as immune system rejection and reduced protein expression efficiency, and future research should focus on developing novel methods to overcome these disadvantages.

## Figures and Tables

**Table 1:** LncRNA as Oncogene in Hepatocellular Carcinoma

Name	Genomic Location	Expression	Targets	Biological Functions in HCC	References
GHET1	7q36.1	Up	ATF1/ KLF2	Predict prognosis and promote tumorigenesis, proliferation.	11, 12
LASP1‐AS	17q12	Up	LASP1	Promote tumorigenesis	13
MALAT1	11q13.1	Up	miR-30a-5p; miR-195/EGFR; miR-143-3p/ZEB1; miR -216b/HIF-2a	Promote tumorigenesis, metastasis and progression Promote chemotherapy resistance; Predictor for Recurrence	14-18
HULC	6p24.3	Up	miR-186/HMGA2; ERK/YB-1; Sirt1	Promote tumorigenesis, progression; Metastasis and predict prognosis; Promote chemotherapy resistance; Tumor diagnosis.	19, 20
HOTAIR	12q13.13	Up	EZH2/miR-122; miR-218/Bmi-1; RNA binding motif protein 38; GLUT1/mTOR.	Promote tumorigenesis, migration and invasion	21-24
ZFAS1	20q13.13	Up	miR-150	Associate with tumor metastasis	25
NEAT1	11q13.1	Up	miR-139/TGF-β1; miR-485/STAT3; MiR-384; miR-101-3p/WEE1; miR-335-c-Met	Promote tumor progression and metastasis; Promote resistance to chemotherapy and radiothererapy	26-28
CRNDE	16q12.2	Up	miR-217/MAPK1; miR-136-5P/IRX5; Wnt/β-catenin	Associate with tumor proliferation, migration, invasion and EMT	30-32
CDKN2B-AS1	9p21.3	Up	let-7c/5p /NAP1L1	Promote tumor growth and metastasis	33
TUG1	22q12.2	Up	miR-455-3p; miR-144/AK2/STAT3; miR-142-3p/ZEB1; KLF2.	Promote tumor proliferation, migration, invasion and induce cell apoptosis	34-37
PVT1	8q24.21	Up	miR-150/HIG2; EZH2/miR-214	Promote tumor invasion, metastasis and predict prognosis	38-41
ZNF667	19q13.43	Up	Bcl-2/BAX	Associate with tumor progression and inhibit apoptosis	42
FAL1	Chromosome IV	Up	miR-1236	Promote tumor proliferation and migration,	43
SNHG12	1p35.3	Up	miR-199a/b-5p	Promote tumorigenesis and metastasis	44
SNHG8	4q26	Up	miR-149-5p	Promote tumorigenesis, metastasis and predict tumor recurrence	45
FEZF1‑AS1	7q31.32	Up	miR‑4443	Contribute to tumor proliferation, migration and invasion	45
SOX21-AS1	13q32.1	Up	p21	Associate with tumor progression and predict prognosis	46
PTTG3P	8q13.1	Up	PTTG1/PI3K/AKT	Promote cell growth, metastasis and activate PI3K/AKT signaling pathway	47
NORAD	20q11.23	Up	miR-202-5p/ TGF-β	Promote tumor progression	48
CASC11	8q24.21	Up	PTEN and PI3K/AKT	Promote tumor migration, invasion and EMT	49
SNHG5	6q14.3	Up	miR-26a-5p/GSK3β	Promote tumor progression	50
CCAT2	8q24.21	Up	NDRG1	promote tumor proliferation and metastasis	51
FOXD2-AS1	1p33	Up	DKK1 and Wnt/β-catenin	promote tumor progression and activate Wnt/β-catenin signaling pathway	52, 169
PAPAS	15q24.3	Up	miR-188-5p	promote tumor progression	53

**Table 2:** LncRNA as Tumor Suppressor in Hepatocellular Carcinoma

Name	Genomic Location	Expression	Targets	Biological Functions in HCC	References
CASC2	10q26.11	Down	miR-24-3p; miR-367/FBXW7; miR-362-5p/NF-kB	Inhibit tumor growth, migration, invasion and EMT	57, 58, 60, 170
DGCR5	22q11.21	Down	miR-346/KLF14; β-catenin/cyclin D1/GSK-3β	Inhibit tumor progression and associate with prognosis	61-63
MEG3	14q32.2	Down	miRNA-664/ADH4; p53	Inhibit tumor progression and associate with prognosis	64, 65
GAS5	17p13.3	Down	miR-135b/RECK/ MMP-2; miR-182/ANGPTL1; miR-21	Inhibit tumor proliferation, migration, invasion and induce cell apoptosis and associate with prognosis	66-68
MIR22HG	17p13.39	Down	miRNA-10a-5p/NCOR2	Inhibit tumor growth, migration, invasion and predict prognosis	69
FENDRR	12q13.13	Down	GPC3	Promote tumorigenesis, migration and invasion	70
HOTAIRM1	7p15.2	Down	Wnt	Inhibit tumor progression	72
AGI2-AS3	7q21.11	Down	miR-374b-5p/SMG1	Inhibit tumor proliferation and migration	73
NRON	q33.3; 9	Down	interleukin-6/STAT3	suppresses tumor cell proliferation and metastasis	74
miR503HG	Xq26.3	Down	HNRNPA2B1/NF-κB	Inhibits tumor metastasis	75

## Abbreviations

HCC: hepatocellular carcinoma
lncRNA: long non-coding RNA
LT: liver transplantation
LR: liver resection
HOTTIP: HOXA transcript at the distal tip
SNP: single nucleotide polymorphisms
MVIH: lncRNA associated with microvascular invasion in hepatocellular carcinoma
NEAT1: nuclear paraspeckle assembly transcript 1
OS: overall survival
MVI: microvascular invasion
MALAT1: metastasis associated lung adenocarcinoma transcript 1
HOTAIR: HOX transcript antisense RNA
EMT: epithelial-mesenchymal transition
linc-ROR: long noncoding RNA regulator of reprogramming
SUMO1P3: SUMO1 Pseudogene 3
H19: H19 imprinted maternally expressed transcript
lncRNA TP73-AS1: LncRNA P73 antisense RNA 1T
FU: fluorouracil
KARL: keap1 regulation-associated lncRNA
CDDP: cisplatin
NRAL: Nrf2 regulation-associated lncRNA
TPTEP1: TPTE Pseudogene 1
NR2F1-AS1: NR2F1 antisense RNA 1
HULC: highly up-regulated in liver cancer
HANR: HCC associated long non-coding RNA
lncARS: lncRNA regulator of akt signaling associated with HCC And RCC
RCC: renal cell carcinoma
SNHG1: small nucleolar RNA host gene 1
SNHG16: small nucleolar RNA host gene 16
SOR: sorafenib
SNHG3: small nucleolar RNA host gene 3
TUC338: transcribed ultra-conserved region 338
TIL: tumor infiltrated lymphocytes
Treg cells: T regulatory cells
AFP: alpha fetal protein
lnc-Tim-3: lnc-T cell immunoglobulin mucin 3
lnc- EGFR: lnc-epidermal growth factor receptor
FENDRR: fetal-lethal non-coding developmental regulatory RNA
Cox-2: cyclooxygenase-2
ROC: receiver operator characteristic
AUC: area under the curve
DCP: des-γ-carboxy prothrombin
LRB1: lncRNA uc007biz.1
UCA1: urothelial cancer associated 1
AAV: adeno-associated viral vector

## References

[1] Bray F, Ferlay J, Soerjomataram I, Siegel RL, Torre LA, Jemal A (2018). Global cancer statistics 2018: GLOBOCAN estimates of incidence and mortality worldwide for 36 cancers in 185 countries. CA Cancer J Clin.

[2] Kulik L, El-Serag HB (2019). Epidemiology and Management of Hepatocellular Carcinoma. Gastroenterology.

[3] Forner A, Reig M, Bruix J (2018). Hepatocellular carcinoma. Lancet.

[4] Attwa MH, El-Etreby SA (2015). Guide for diagnosis and treatment of hepatocellular carcinoma. World J Hepatol.

[5] Liang Q, Shen XY, Sun GC (2018). Precision medicine: Update on diagnosis and therapeutic strategies of hepatocellular carcinoma. Curr Med Chem.

[6] Lepage C, Capocaccia R, Hackl M, Lemmens V, Molina E, Pierannunzio D (2015). Survival in patients with primary liver cancer, gallbladder and extrahepatic biliary tract cancer and pancreatic cancer in Europe 1999-2007: Results of EUROCARE-5. Eur J Cancer.

[7] Ulitsky I, Bartel DP (2013). lincRNAs: genomics, evolution, and mechanisms. Cell.

[8] ENCODE Project Consortium (2012). An integrated encyclopedia of DNA elements in the human genome. Nature.

[9] Sun M, Kraus WL (2015). From discovery to function: the expanding roles of long noncoding RNAs in physiology and disease. Endocr Rev.

[10] Dhamija S, Diederichs S (2016). From junk to master regulators of invasion: lncRNA functions in migration, EMT and metastasis. Int J Cancer.

[11] Ding GQ, Li W, Liu JP, Zeng YL, Mao CS, Kang Y (2017). LncRNA GHET1 activated by H3K27 acetylation promotes cell tumorigenesis through regulating ATF1 in hepatocellular carcinoma. Biomed Pharmacother.

[12] Zhu YF, Tong Y, Wu J, Liu YJ, Zhao MJ (2019). Knockdown of LncRNA GHET1 suppresses prostate cancer cell proliferation by inhibiting HIF-1α/Notch-1 signaling pathway via KLF2. Biofactors.

[13] Yin L, Chen YH, Zhou Y, Deng GL, Han Y, Guo C (2019). Increased long noncoding RNA LASP1-AS is critical for hepatocellular carcinoma tumorigenesis via upregulating LASP1. J Cell Physiol.

[14] Malakar P, Stein I, Saragovi A, Winkler R, Stern-Ginossar N, Berger M (2019). Long Noncoding RNA MALAT1 regulates cancer glucose metabolism by enhancing mTOR-mediated translation of TCF7L2. Cancer Res.

[15] Chen L S, Yao HB, Wang K, Liu XF (2017). Long Non-Coding RNA MALAT1 regulates ZEB1 expression by sponging miR-143-3p and promotes hepatocellular carcinoma progression. J Cell Biochem.

[16] Yuan P, Cao WB, Zang QL, Li GX, Guo XF, Fan JH (2016). The HIF-2α-MALAT1-miR-216b axis regulates multi-drug resistance of hepatocellular carcinoma cells via modulating autophagy. Biochem Biophys Res Commun.

[17] Pan YJ, Tong SM, Cui RJ, Fan JL, Liu C, Lin YC (2018). Long Non-Coding MALAT1 functions as a competing endogenous RNA to regulate vimentin expression by sponging miR-30a-5p in hepatocellular carcinoma. Cell Physiol Biochem.

[18] Liu DL, Zhu Y, Pang JK, Weng X, Feng XR, Guo YB (2018). Knockdown of long non-coding RNA MALAT1 inhibits growth and motility of human hepatoma cells via modulation of miR-195. J Cell Biochem.

[19] Wang Y, Chen FQ, Zhao M, Yang Z, Li J, Zhang SQ (2017). The long noncoding RNA HULC promotes liver cancer by increasing the expression of the HMGA2 oncogene via sequestration of the microRNA-186. J Biol Chem.

[20] Li D, Liu XF, Zhou J, Hu J, Zhang DD, Liu J (2017). Long noncoding RNA HULC modulates the phosphorylation of YB-1 through serving as a scaffold of extracellular signal-regulated kinase and YB-1 to enhance hepatocarcinogenesis. Hepatology.

[21] Wei SB, Fan Q, Yang L, Zhang XD, Ma YB, Zong ZH (2017). Promotion of glycolysis by HOTAIR through GLUT1 upregulation via mTOR signaling. Oncol Rep.

[22] Ding CF, Cheng SB, Yang Z, Lv Z, Xiao H, Du CL (2014). Long non-coding RNA HOTAIR promotes cell migration and invasion via down-regulation of RNA binding motif protein 38 in hepatocellular carcinoma cells. Int J Mol Sci.

[23] Cheng D, Deng JG, Zhang B, He XY, Meng Z, Li GL (2018). LncRNA HOTAIR epigenetically suppresses miR-122 expression in hepatocellular carcinoma via DNA methylation. EBioMedicine.

[24] Fu WM, Zhu X, Wang WM, Lu YF, Hu BG, Wang H (2015). Hotair mediates hepatocarcinogenesis through suppressing miRNA-218 expression and activating P14 and P16 signaling. J Hepatol.

[25] Li T, Xie JJ, Shen C, Cheng DF, Shi Y, Wu ZC (2015). Amplification of long non coding RNA ZFAS1 promotes metastasis in hepatocellular carcinoma. Cancer Res.

[26] Tu JF, Zhao ZW, Xu M, Lu XJ, Chang L, Ji JS (2018). NEAT1 upregulates TGF-β1 to induce hepatocellular carcinoma progression by sponging hsa-mir-139-5p. J Cell Physiol.

[27] Zhang XN, Zhou J, Lu XJ (2018). The long noncoding RNA NEAT1 contributes to hepatocellular carcinoma development by sponging miR-485 and enhancing the expression of the STAT3. J Cell Physiol.

[28] Zhu LY, Yang NY, Li CC, Liu GQ, Pan W, Li X (2018). Long noncoding RNA NEAT1 promotes cell proliferation, migration, and invasion in hepatocellular carcinoma through interacting with miR-384. J Cell Biochem.

[29] Yan K, Fu Y, Zhu N, Wang Z, Hong JL, Li Y (2019). Repression of lncRNA NEAT1 enhances the antitumor activity of CD8(+)T cells against hepatocellular carcinoma via regulating miR-155/Tim-3. Int J Biochem Cell Biol.

[30] Wang HH, Ke J, Guo QN, Nampoukime KB, Yang PW, Ma K (2018). Long non-coding RNA CRNDE promotes the proliferation, migration and invasion of hepatocellular carcinoma cells through miR-217/MAPK1 axis. J Cell Mol Med.

[31] Zhu LY, Liu YY, Chen QX, Yu GF, Chen J, Chen K (2018). Long-Noncoding RNA colorectal neoplasia differentially expressed gene as a potential target to uregulate the expression of IRX5 by miR-136-5P to promote oncogenic properties in hepatocellular carcinoma. Cell Physiol Biochem.

[32] Zhu LY, Yang NY, Du GQ, Li CC, Liu GQ, Liu SJ (2018). LncRNA CRNDE promotes the epithelial-mesenchymal transition of hepatocellular carcinoma cells via enhancing the Wnt/β-catenin signaling pathway. J Cell Biochem.

[33] Huang YQ, Xiang B, Liu YH, Wang Y, Kan HP (2018). LncRNA CDKN2B-AS1 promotes tumor growth and metastasis of human hepatocellular carcinoma by targeting let-7c-5p/NAP1L1 axis. Cancer Lett.

[34] Lv J, Kong YK, Gao ZQ, Liu YM, Zhu PF, Yu ZJ (2018). LncRNA TUG1 interacting with miR-144 contributes to proliferation, migration and tumorigenesis through activating the JAK2/STAT3 pathway in hepatocellular carcinoma. Int J Biochem Cell Biol.

[35] He C, Liu ZG, Jin L, Zhang F, Peng XH, Xiao YQ (2018). lncRNA TUG1-mediated Mir-142-3p downregulation contributes to metastasis and the epithelial-to-mesenchymal transition of hepatocellular carcinoma by targeting ZEB1. Cell Physiol Biochem.

[36] Huang MD, Chen WM, Qi FZ (2015). Long non-coding RNA TUG1 is up-regulated in hepatocellular carcinoma and promotes cell growth and apoptosis by epigenetically silencing of KLF2. Mol Cancer.

[37] Lin YH, Wu MH, Huang YH, Yeh CT, Cheng ML, Chi HC (2018). Taurine up-regulated gene 1 functions as a master regulator to coordinate glycolysis and metastasis in hepatocellular carcinoma. Hepatology.

[38] Ding CF, Yang Z, Lv Z, Du CL, Xiao H, Peng CH (2015). Long non-coding RNA PVT1 is associated with tumor progression and predicts recurrence in hepatocellular carcinoma patients. Oncol Lett.

[39] Xu YXX, Luo XX, He WG, Chen GC, Li YS, Li WX (2018). Long non-coding RNA PVT1/miR-150/ HIG2 axis regulates the proliferation, invasion and the balance of iron metabolism of hepatocellular carcinoma. Cell Physiol Biochem.

[40] Guo JP, Hao C, Wang CC, Li L (2018). Long noncoding RNA PVT1 modulates hepatocellular carcinoma cell proliferation and apoptosis by recruiting EZH2. Cancer Cell Int.

[41] Gou X, Zhao XY, Wang ZR (2017). Long noncoding RNA PVT1 promotes hepatocellular carcinoma progression through regulating miR-214. Cancer Biomark.

[42] Cheng K, Chen ZZ, Liu L, Zhao YJ, Zhang S, Wang Q (2017). ZNF667 serves as a putative oncogene in human hepatocellular carcinoma. Cell Physiol Biochem.

[43] Li BG, Mao R, Liu CF, Zhang WH, Tang Y, Guo Z (2018). LncRNA FAL1 promotes cell proliferation and migration by acting as a CeRNA of miR-1236 in hepatocellular carcinoma cells. Life Sci.

[44] Lan T, Ma WJ, Hong ZF, Wu L, Chen X, Yuan YF (2017). Long non-coding RNA small nucleolar RNA host gene 12 (SNHG12) promotes tumorigenesis and metastasis by targeting miR-199a/b-5p in hepatocellular carcinoma. J Exp Clin Cancer Res.

[45] Dong JY, Teng F, Guo WY, Yang JH, Ding GS, Fu ZR (2018). lncRNA SNHG8 promotes the tumorigenesis and metastasis by sponging miR-149-5p and predicts tumor recurrence in hepatocellular carcinoma. Cell Physiol Biochem.

[46] Wei CX, Wang H, Xu F, Liu Z, Jiang RD (2018). LncRNA SOX21-AS1 is associated with progression of hepatocellular carcinoma and predicts prognosis through epigenetically silencing p21. Biomed Pharmacother.

[47] Huang JL, Cao SW, Ou QS, Yang B, Zheng SH, Tang J (2018). The long non-coding RNA PTTG3P promotes cell growth and metastasis via up-regulating PTTG1 and activating PI3K/AKT signaling in hepatocellular carcinoma. Mol Cancer.

[48] Yang X, Cai JB, Peng R, Wei CY, Lu JC, Gao C (2019). The long noncoding RNA NORAD enhances the TGF-β pathway to promote hepatocellular carcinoma progression by targeting miR-202-5p. J Cell Physiol.

[49] Han YD, Chen MZ, Wang AL, Fan XP (2019). STAT3-induced upregulation of lncRNA CASC11 promotes the cell migration, invasion and epithelial-mesenchymal transition in hepatocellular carcinoma by epigenetically silencing PTEN and activating PI3K/AKT signaling pathway. Biochem Biophys Res Commun.

[50] Li YR, Guo D, Zhao Y, Ren MD, Lu GF, Wang Y (2018). Long non-coding RNA SNHG5 promotes human hepatocellular carcinoma progression by regulating miR-26a-5p/GSK3β signal pathway. Cell Death Dis.

[51] Liu YY, Wang D, Li YG, Yan SY, Dang H, Yue H (2019). Long noncoding RNA CCAT2 promotes hepatocellular carcinoma proliferation and metastasis through up-regulation of NDRG1. Exp Cell Res.

[52] Chang YH, Zhang J, Zhou CC, Qiu GL, Wang GH, Wang SF (2018). Long non-coding RNA FOXD2-AS1 plays an oncogenic role in hepatocellular carcinoma by targeting miR-206. Oncol Rep.

[53] Ma JC, Qin CY, Yuan ZG, Liu SL (2019). LncRNA PAPAS promotes hepatocellular carcinoma by interacting with miR-188-5p. J Cell Biochem.

[54] Ji J, Yin Y, Ju HY, Xu XL, Liu W, Fu Q (2018). Long non-coding RNA Lnc-Tim3 exacerbates CD8 T cell exhaustion via binding to Tim-3 and inducing nuclear translocation of bat3 in HCC. Cell Death Dis.

[55] Jiang RQ, Tang JW, Chen Y, Deng L, Ji J, Xie Y (2017). The long noncoding RNA lnc-EGFR stimulates T-regulatory cells differentiation thus promoting hepatocellular carcinoma immune evasion. Nat Commun.

[56] Li X, Lei Y, Wu M, Li N (2018). Regulation of mcrophage activation and polarization by HCC-derived exosomal lncRNA TUC339. Int J Mol Sci.

[57] Fan JC, Zeng F, Le YG, Xin L (2018). LncRNA CASC2 inhibited the viability and induced the apoptosis of hepatocellular carcinoma cells through regulating miR-24-3p. J Cell Biochem.

[58] Wang YF, Liu ZK, Yao BW, Li Q, Wang L, Wang C (2017). Long non-coding RNA CASC2 suppresses epithelial-mesenchymal transition of hepatocellular carcinoma cells through CASC2/miR-367/FBXW7 axis. Mol Cancer.

[59] Liu ZC, Dang CS, Xing ET, Zhao MJ, Shi LC, Sun JW (2019). Overexpression of CASC2 improves cisplatin sensitivity in hepatocellular carcinoma through sponging miR-222. DNA Cell Biol.

[60] Zhao L, Zhang YJ, Zhang YB (2018). Long noncoding RNA CASC2 regulates hepatocellular carcinoma cell oncogenesis through miR-362-5p/Nf-κB axis. J Cell Physiol.

[61] Wang YG, Liu J, Shi M, Chen FX (2018). LncRNA DGCR5 represses the development of hepatocellular carcinoma by targeting the miR-346/KLF14 axis. J Cell Physiol.

[62] Huang RY, Wang XC, Zhang WJ, Zhangyuan GY, Jin KP (2016). Down-regulation of LncRNA DGCR5 correlates with poor prognosis in hepatocellular carcinoma. Cell Physiol Biochem.

[63] Wang XL, Shi M, Xiang T, Bu YZ (2019). Long noncoding RNA DGCR5 represses hepatocellular carcinoma progression by inactivating Wnt signaling pathway. J Cell Biochem.

[64] He JH, Han ZP, Liu JM, Zhou JB, Zou MX, Lv YB (2017). Overexpression of long non-coding RNA MEG3 inhibits proliferation of hepatocellular carcinoma Huh7 cells via negative modulation of miRNA-664. J Cell Biochem.

[65] Zhu JJ, Liu SS, Ye FQ, Shen Y, Tie Y, Zhu J (2015). Long noncoding RNA MEG3 interacts with p53 protein and regulates partial p53 target genes in hepatoma cells. PLoS One.

[66] Yang L, Jiang JS (2019). GAS5 regulates RECK expression and inhibits invasion potential of HCC cells by sponging miR-135b. Biomed Res Int.

[67] Chang L, Li CC, Lan T, Wu L, Yuan YF, Liu QY (2016). Decreased expression of long non-coding RNA GAS5 indicates a poor prognosis and promotes cell proliferation and invasion in hepatocellular carcinoma by regulating vimentin. Mol Med Rep.

[68] Hu LT, Ye H, Huang GM, Luo F, Liu YW, Liu Y (2016). Long noncoding RNA GAS5 suppresses the migration and invasion of hepatocellular carcinoma cells via miR-21. Tumour Biol.

[69] Dong Y, Yan WW, Zhang SL, Zhang MZH, Zhou YP, Ling HH (2017). Prognostic values of long non-coding RNA MIR22HG for patients with hepatocellular carcinoma after hepatectomy. Oncotarget.

[70] Wang B, Xian JC, Zang JF, Xiao L, Li Y, Sha M (2019). Long non-coding RNA FENDRR inhibits proliferation and invasion of hepatocellular carcinoma by down-regulating glypican-3 expression. Biochem Biophys Res Commun.

[71] Yu ZY, Zhao H, Feng X, Li HB, Qiu CH, Yi XM (2019). Long Non-coding RNA FENDRR acts as a miR-423-5p sponge to suppress the Treg-mediated immune escape of hepatocellular carcinoma cells. Mol Ther Nucleic Acids.

[72] Zhang Y, Mi L, Xuan Y, Gao C, Wang YH, Ming HX (2018). LncRNA HOTAIRM1 inhibits the progression of hepatocellular carcinoma by inhibiting the Wnt signaling pathway. Eur Rev Med Pharmacol Sci.

[73] Yin Z, Ma TT, Yan JH, Shi N, Zhang CZ, Lu X (2019). LncRNA MAGI2-AS3 inhibits hepatocellular carcinoma cell proliferation and migration by targeting the miR-374b-5p/SMG1 signaling pathway. J Cell Physiol.

[74] Yao ZC, Xiong ZY, Li RX, Liang H, Jia CC, Deng MH (2018). Long non-coding RNA NRON is downregulated in HCC and suppresses tumour cell proliferation and metastasis. Biomed Pharmacother.

[75] Wang H, Liang LH, Dong QZ, Huan L, He J, Li BT (2018). Long noncoding RNA miR503HG, a prognostic indicator, inhibits tumor metastasis by regulating the HNRNPA2B1/NF-κB pathway in hepatocellular carcinoma. Theranostics.

[76] Ye YB, Xu YXX, Lai Y, He WG, Li YS, Wang RM (2018). Long non-coding RNA cox-2 prevents immune evasion and metastasis of hepatocellular carcinoma by altering M1/M2 macrophage polarization. J Cell Biochem.

[77] Zhou L, Liu J, Luo F (2006). Serum tumor markers for detection of hepatocellular carcinoma. World J Gastroenterol.

[78] Xu H, Chen YM, Dong XY, Wang XJ (2018). Serum exosomal long noncoding RNAs ENSG00000258332.1 and LINC00635 for the diagnosis and prognosis of hepatocellular carcinoma. Cancer Epidemiol Biomarkers Prev.

[79] Gao SB, Xu XH, Wang Y, Zhang W, Wang XY (2018). Diagnostic utility of plasma lncRNA small nucleolar RNA host gene 1 in patients with hepatocellular carcinoma. Mol Med Rep.

[80] Li J, Wang XC, Tang JW, Jiang RQ, Zhang WJ, Ji J (2015). HULC and Linc00152 act as novel biomarkers in predicting diagnosis of hepatocellular carcinoma. Cell Physiol Biochem.

[81] Wang ZF, Hu R, Pang JM, Zhang GZ, Yan W, Li ZN (2018). Serum long noncoding RNA LRB1 as a potential biomarker for predicting the diagnosis and prognosis of human hepatocellular carcinoma. Oncol Lett.

[82] Zheng ZK, Pang C, Yang Y, Duan Q, Zhang J, Liu WC (2018). Serum long noncoding RNA urothelial carcinoma-associated 1: A novel biomarker for diagnosis and prognosis of hepatocellular carcinoma. J Int Med Res.

[83] Wu LM, Yang Z, Zhang J, Xie HY, Zhou L, Zheng SS (2018). Long noncoding RNA HOTTIP expression predicts tumor recurrence in hepatocellular carcinoma patients following liver transplantation. Hepatobiliary Surg Nutr.

[84] Yuan SX, Yang F, Yang Y, Tao QF, Zhang J, Huang G (2012). Long noncoding RNA associated with microvascular invasion in hepatocellular carcinoma promotes angiogenesis and serves as a predictor for hepatocellular carcinoma patients' poor recurrence-free survival after hepatectomy. Hepatology.

[85] Zhang JL, Zhao BC, Chen XX, Wang ZN, Xu HM, Huang BJ (2018). Silence of long noncoding RNA NEAT1 inhibits malignant biological behaviors and chemotherapy resistance in gastric cancer. Pathol Oncol Res.

[86] Liu Z, Chang Q, Yang F, Liu B, Yao HW, Bai ZG (2017). Long non-coding RNA NEAT1 overexpression is associated with unfavorable prognosis in patients with hepatocellular carcinoma after hepatectomy: A Chinese population-based study. Eur J Surg Oncol.

[87] Sun YT, Ma L (2019). New Insights into Long Non-Coding RNA MALAT1 in Cancer and Metastasis. Cancers (Basel).

[88] Lai MC, Yang Z, Zhou L, Zhu QQ, Xie HY, Zhang F (2012). Long non-coding RNA MALAT-1 overexpression predicts tumor recurrence of hepatocellular carcinoma after liver transplantation. Med Oncol.

[89] Hajjari M, Salavaty A (2015). HOTAIR: an oncogenic long non-coding RNA in different cancers. Cancer Biol Med.

[90] Yang Z, Zhou L, Wu LM, Lai MC, Xie HY, Zhang F (2011). Overexpression of long non-coding RNA HOTAIR predicts tumor recurrence in hepatocellular carcinoma patients following liver transplantation. Ann Surg Oncol.

[91] Marx V (2014). Cancer treatment: Sharp shooters. Nature.

[92] Wu J, Li Y, Dang YZ, Gao HX, Jiang LJ, Chen ZN (2015). HAb18G/CD147 promotes radioresistance in hepatocellular carcinoma cells: a potential role for integrin β1 signaling. Mol Cancer Ther.

[93] Chen X, Zhang NB (2019). Downregulation of lncRNA NEAT1_2 radiosensitizes hepatocellular carcinoma cells through regulation of miR-101-3p/WEE1 axis. Cell Biol Int.

[94] Sharma A, Singh K, Almasan A (2012). Histone H2AX phosphorylation: a marker for DNA damage. Methods Mol Biol.

[95] Yang Y, Gao YZ, Mutter-Rottmayer L, Zlatanou A, Durando M, Ding WM (2017). DNA repair factor RAD18 and DNA polymerase Polκ confer tolerance of oncogenic DNA replication stress. J Cell Biol.

[96] Chen Y, Shen ZT, Zhi YG, Zhou H, Zhang K, Wang T (2018). Long non-coding RNA ROR promotes radioresistance in hepatocelluar carcinoma cells by acting as a ceRNA for microRNA-145 to regulate RAD18 expression. Arch Biochem Biophys.

[97] Chen Y, Shen ZT, Zhi YG, Zhou H, Zhang K, Wang T (2013). Up-regulation of SUMO1 pseudogene 3 (SUMO1P3) in gastric cancer and its clinical association. Med Oncol.

[98] Zhan YH, Liu YC, Wang CL, Lin JH, Chen MW, Chen XY (2016). Increased expression of SUMO1P3 predicts poor prognosis and promotes tumor growth and metastasis in bladder cancer. Oncotarget.

[99] Zhang YW, Li Y, Han L, Zhang PY, Sun SY (2019). SUMO1P3 is associated clinical progression and facilitates cell migration and invasion through regulating miR-136 in non-small cell lung cancer. Biomed Pharmacother.

[100] Zhou Y, He P, Xie XH, Sun CY (2019). Knockdown of SUMO1P3 represses tumor growth and invasion and enhances radiosensitivity in hepatocellular carcinoma. Mol Cell Biochem.

[101] Poirier F, Chan CT, Timmons PM, Robertson EJ, Evans MJ, Rigby PW (1991). The murine H19 gene is activated during embryonic stem cell differentiation *in vitro* and at the time of implantation in the developing embryo. Development.

[102] Matouk IJ, DeGroot N, Mezan S, Ayesh S, Abu-lail R, Hochberg A (2007). The H19 non-coding RNA is essential for human tumor growth. PLoS One.

[103] Ma HB, Yuan L, Li WH, Xu KY, Yang L (2018). The LncRNA H19/miR-193a-3p axis modifies the radio-resistance and chemotherapeutic tolerance of hepatocellular carcinoma cells by targeting PSEN1. J Cell Biochem.

[104] Zhang YH, Huang B, Chen Z, Yang SM (2020). Knockdown of LINC00473 enhances radiosensitivity in hepatocellular carcinoma via regulating the miR-345-5p/FOXP1 Axis. Onco Targets Ther.

[105] Wong KY, Li ZH, Zhang XQ, Leung GKK, Chan GCF, Chim CS (2015). Epigenetic silencing of a long non-coding RNA KIAA0495 in multiple myeloma. Mol Cancer.

[106] Li SL, Huang Y, Huang Y, Fu YM, Tang DL, Kang R (2017). The long non-coding RNA TP73-AS1 modulates HCC cell proliferation through miR-200a-dependent HMGB1/RAGE regulation. J Exp Clin Cancer Res.

[107] Ma CX, Gao WC, Tian L (2019). LncRNA TP73-AS1 promotes malignant progression of hepatoma by regulating microRNA-103. Eur Rev Med Pharmacol Sci.

[108] Song W, Zhang JJ, Xia QX, Sun MM (2019). Down-regulated lncRNA TP73-AS1 reduces radioresistance in hepatocellular carcinoma via the PTEN/Akt signaling pathway. Cell Cycle.

[109] Forner A, Gilabert M, Bruix J, Raoul JL (2014). Treatment of intermediate-stage hepatocellular carcinoma. Nat Rev Clin Oncol.

[110] Le Grazie M, Biagini MR, Tarocchi M, Polvani S, Galli A (2017). Chemotherapy for hepatocellular carcinoma: The present and the future. World J Hepatol.

[111] Lohitesh K, Chowdhury R, Mukherjee S (2018). Resistance a major hindrance to chemotherapy in hepatocellular carcinoma: an insight. Cancer Cell Int.

[112] Wu LL, Pan CW, Wei X, Shi YF, Zheng JJ, Lin XY (2018). lncRNA KRAL reverses 5-fluorouracil resistance in hepatocellular carcinoma cells by acting as a ceRNA against miR-141. Cell Commun Signal.

[113] Shi L, Wu LL, Chen ZG, Yang JQ, Chen XF, Yu FY (2015). MiR-141 activates Nrf2-dependent antioxidant pathway via down-regulating the expression of Keap1 conferring the resistance of hepatocellular carcinoma cells to 5-Fluorouracil. Cell Physiol Biochem.

[114] Tsuchida K, Tsujita T, Hayashi M, Ojima A, Keleku-Lukwete N, Katsuoka F (2017). Halofuginone enhances the chemo-sensitivity of cancer cells by suppressing NRF2 accumulation. Free Radic Biol Med.

[115] van Jaarsveld MT, Helleman J, Boersma AW, van Kuijk PF, van Ijcken WF, Despierre E (2013). miR-141 regulates KEAP1 and modulates cisplatin sensitivity in ovarian cancer cells. Oncogene.

[116] Ji P, Diederichs S, Wang W, Böing S, Metzger R, Schneider PM (2003). MALAT-1, a novel noncoding RNA, and thymosin beta4 predict metastasis and survival in early-stage non-small cell lung cancer. Oncogene.

[117] Zhang XM, Hamblin MH, Yin KJ (2017). The long noncoding RNA Malat1: Its physiological and pathophysiological functions. RNA Biol.

[118] Sun Q, Hao Q, Prasanth KV (2018). Nuclear long noncoding RNAs: Key regulators of gene expression. Trends Genet.

[119] Lopez-Ayllon BD, Moncho-Amor V, Abarrategi A, Ibañez de Cáceres I, Castro-Carpeño J, Belda-Iniesta C (2014). Cancer stem cells and cisplatin-resistant cells isolated from non-small-lung cancer cell lines constitute related cell populations. Cancer Med.

[120] Schmidt LH, Görlich D, Spieker T, Rohde C, Schuler M, Mohr M (2014). Prognostic impact of Bcl-2 depends on tumor histology and expression of MALAT-1 lncRNA in non-small-cell lung cancer. J Thorac Oncol.

[121] Tee AE, Liu B, Song RH, Li JY, Pasquier E, Cheung BB (2016). The long noncoding RNA MALAT1 promotes tumor-driven angiogenesis by up-regulating pro-angiogenic gene expression. Oncotarget.

[122] Fan TT, Zhang CS, Zong M, Zhao QD, Yang X, Hao C (2014). Peptidylarginine deiminase IV promotes the development of chemoresistance through inducing autophagy in hepatocellular carcinoma. Cell Biosci.

[123] Mazzanti R, Platini F, Bottini C, Fantappiè O, Solazzo M, Tessitore L (2009). Down-regulation of the HGF/MET autocrine loop induced by celecoxib and mediated by P-gp in MDR-positive human hepatocellular carcinoma cell line. Biochem Pharmacol.

[124] Wu LL, Cai WP, Lei X, Shi KQ, Lin XY, Shi L (2019). NRAL mediates cisplatin resistance in hepatocellular carcinoma via miR-340-5p/Nrf2 axis. J Cell Commun Signal.

[125] Li ZH, Wong KY, Calin GA, Chng WJ, Chan GC, Chim CS (2019). Epigenetic silencing of miR-340-5p in multiple myeloma: mechanisms and prognostic impact. Clin Epigenetics.

[126] Schneider C, King RM, Philipson L (1988). Genes specifically expressed at growth arrest of mammalian cells. Cell.

[127] Zhao PX, Cui X, Zhao LY, Liu L, Wang DY (2020). Overexpression of growth-arrest-specific transcript 5 improved cisplatin sensitivity in hepatocellular carcinoma through sponging miR-222. DNA Cell Biol.

[128] Guo LY, Peng Y, Meng YY, Liu YD, Yang SS, Jin H (2017). Expression profiles analysis reveals an integrated miRNA-lncRNA signature to predict survival in ovarian cancer patients with wild-type BRCA1/2. Oncotarget.

[129] Chen YH, Zhao H, Li HB, Feng X, Tang H, Qiu CH (2020). LINC01234/MicroRNA-31-5p/MAGEA3 axis mediates the proliferation and chemoresistance of hepatocellular carcinoma cells. Molecular therapy. Nucleic acids.

[130] Liang QY, Ding JY, Xu RZ, Xu ZF, Zheng S (2010). The novel human endogenous retrovirus-related gene, psiTPTE22-HERV, is silenced by DNA methylation in cancers. Int J Cancer.

[131] Ding HD, Liu JP, Zou RY, Cheng PR, Su Y (2019). Long non-coding RNA TPTEP1 inhibits hepatocellular carcinoma progression by suppressing STAT3 phosphorylation. Journal of experimental & clinical cancer research: CR.

[132] Huang H, Chen J, Ding CM, Jin X, Jia ZM, Peng J (2018). LncRNA NR2F1-AS1 regulates hepatocellular carcinoma oxaliplatin resistance by targeting ABCC1 via miR-363. J Cell Mol Med.

[133] Panzitt K, Tschernatsch MM, Guelly C, Moustafa T, Stradner M, Strohmaier HM (2007). Characterization of HULC, a novel gene with striking up-regulation in hepatocellular carcinoma, as noncoding RNA. Gastroenterology.

[134] Xiong H, Ni Z, He J, Jiang S, Li X, He J (2017). LncRNA HULC triggers autophagy via stabilizing Sirt1 and attenuates the chemosensitivity of HCC cells. Oncogene.

[135] Yu X, Zheng HY, Chan MT, Wu WK (2017). HULC: an oncogenic long non-coding RNA in human cancer. J Cell Mol Med.

[136] Wang ZQ, Chen WY (2013). Emerging roles of SIRT1 in cancer drug resistance. Genes Cancer.

[137] Xiao J, Lv Y, Jin FJ, Liu YX, Ma Y, Xiong YJ (2017). LncRNA HANR promotes tumorigenesis and increase of chemoresistance in hepatocellular carcinoma. Cell Physiol Biochem.

[138] Domoto T, Pyko IV, Furuta T, Miyashita K, Uehara M, Shimasaki T (2016). Glycogen synthase kinase-3β is a pivotal mediator of cancer invasion and resistance to therapy. Cancer Sci.

[139] Qu L, Ding J, Chen C, Wu ZJ, Liu B, Gao Y (2016). Exosome-transmitted lncARSR promotes sunitinib resistance in renal cancer by acting as a competing endogenous RNA. Cancer Cell.

[140] Li YL, Ye Y, Feng BM, Qi Y (2017). Long noncoding RNA lncARSR promotes doxorubicin resistance in hepatocellular carcinoma via modulating PTEN-PI3K/Akt pathway. J Cell Biochem.

[141] Yuan L, Lv YR, Li HC, Gao HD, Song SS, Zhang Y (2015). Deubiquitylase OTUD3 regulates PTEN stability and suppresses tumorigenesis. Nat Cell Biol.

[142] Llovet JM, Montal R, Sia D, Finn RS (2018). Molecular therapies and precision medicine for hepatocellular carcinoma. Nat Rev Clin Oncol.

[143] Sanoff HK, Chang YK, Lund JL, O'Neil BH, Dusetzina SB (2016). Sorafenib effectiveness in advanced hepatocellular carcinoma. Oncologist.

[144] Zhu YJ, Zheng B, Wang HY, Chen L (2017). New knowledge of the mechanisms of sorafenib resistance in liver cancer. Acta Pharmacol Sin.

[145] Li WD, Dong XS, He CJ, Tan G, Li ZY, Zhai B (2019). LncRNA SNHG1 contributes to sorafenib resistance by activating the Akt pathway and is positively regulated by miR-21 in hepatocellular carcinoma cells. J Exp Clin Cancer Res.

[146] Sun Y, Wei G, Luo H, Wu W, Skogerbø G, Luo J (2017). The long noncoding RNA SNHG1 promotes tumor growth through regulating transcription of both local and distal genes. Oncogene.

[147] Xu CL, Hu CM, Wang YX, Liu S (2019). Long noncoding RNA SNHG16 induces sorafenib resistance in hepatocellular carcinoma cells through sponging miR-140-5p. Onco Targets Ther.

[148] Fang Z, Yin S, Sun RC, Zhang SX, Fu M, Wu Y (2017). miR-140-5p suppresses the proliferation, migration and invasion of gastric cancer by regulating YES1. Mol Cancer.

[149] Zhang PF, Wang F, Wu J, Wu Y, Huang W, Liu D (2019). LncRNA SNHG3 induces EMT and sorafenib resistance by modulating the miR-128/CD151 pathway in hepatocellular carcinoma. J Cell Physiol.

[150] Ke AW, Shi GM, Zhou J, Huang XY, Shi YH, Ding ZB (2011). CD151 amplifies signaling by integrin α6β1 to PI3K and induces the epithelial-mesenchymal transition in HCC cells. Gastroenterology.

[151] Li G, Zhang Y, Mao J, Hu P, Chen Q, Ding W (2019). LncRNA TUC338 is overexpressed in prostate carcinoma and downregulates miR-466. Gene.

[152] Ouyang KX, Zou R, Liang J, Bai ZB, Li ZQ, Zhao JJ (2017). TUC338 overexpression leads to enhanced proliferation and reduced apoptosis in tongue squamous cell carcinoma cells *in vitro*. J Oral Maxillofac Surg.

[153] Jin WD, Chen L, Cai X, Zhang YX, Zhang JX, Ma DD (2017). Long non-coding RNA TUC338 is functionally involved in sorafenib-sensitized hepatocarcinoma cells by targeting RASAL1. Oncol Rep.

[154] Liu DX, Yang CF, Bojdani E, Murugan AK, Xing MZ (2013). Identification of RASAL1 as a major tumor suppressor gene in thyroid cancer. J Natl Cancer Inst.

[155] Chen SW, Xia XH (2019). Long noncoding RNA NEAT1 suppresses sorafenib sensitivity of hepatocellular carcinoma cells via regulating miR-335-c-Met. J Cell Physiol.

[156] Gao Y, Zeng F, Wu JY, Li HY, Fan JJ, Mai L (2015). MiR-335 inhibits migration of breast cancer cells through targeting oncoprotein c-Met. Tumour Biol.

[157] Li XY, Zhou Y, Yang L, Ma YB, Peng XQ, Yang S (2020). LncRNA NEAT1 promotes autophagy via regulating miR-204/ATG3 and enhanced cell resistance to sorafenib in hepatocellular carcinoma. J Cell Physiol.

[158] Aerts M, Benteyn D, Van Vlierberghe H, Thielemans K, Reynaert H (2016). Current status and perspectives of immune-based therapies for hepatocellular carcinoma. World J Gastroenterol.

[159] Komohara Y, Fujiwara Y, Ohnishi K, Takeya M (2016). Tumor-associated macrophages: Potential therapeutic targets for anti-cancer therapy. Adv Drug Deliv Rev.

[160] Kobayashi N, Hiraoka N, Yamagami W, Ojima H, Kanai Y, Kosuge T (2007). FOXP3+ regulatory T cells affect the development and progression of hepatocarcinogenesis. Clin Cancer Res.

[161] Breous E, Thimme R (2011). Potential of immunotherapy for hepatocellular carcinoma. J Hepatol.

[162] Mou YH, Wang DG, Xing RW, Nie HQ, Mou YP, Zhang Y (2018). Identification of long noncoding RNAs biomarkers in patients with hepatitis B virus-associated hepatocellular carcinoma. Cancer Biomark.

[163] Carpenter S, Aiello D, Atianand MK, Ricci EP, Gandhi P, Hall LL (2013). A long noncoding RNA mediates both activation and repression of immune response genes. Science.

[164] Dinarello CA (2010). Anti-inflammatory Agents: Present and Future. Cell.

[165] Goessling W, North TE, Loewer S, Lord AM, Lee S, Stoick-Cooper CL (2009). Genetic interaction of PGE2 and wnt signaling regulates developmental specification of stem cells and regeneration. Cell.

[166] Vegiopoulos A, Müller-Decker K, Strzoda D, Schmitt I, Chichelnitskiy E, Ostertag A (2010). Cyclooxygenase-2 controls energy homeostasis in mice by de novo recruitment of brown adipocytes. Science.

[167] Kogure T, Yan IK, Lin WL, Patel T (2013). Extracellular vesicle-mediated transfer of a novel long noncoding RNA TUC339: A mechanism of intercellular signaling in human hepatocellular cancer. Genes Cancer.

[168] Hou ZH, Xu XW, Fu XY, Zhou LD, Liu SP, Tan DM (2020). Long non-coding RNA MALAT1 promotes angiogenesis and immunosuppressive properties of HCC cells by sponging miR-140. Am J Physiol Cell Physiol.

[169] Lei T, Zhu XD, Zhu K, Jia FX, Li SQ (2019). EGR1-induced upregulation of lncRNA FOXD2-AS1 promotes the progression of hepatocellular carcinoma via epigenetically silencing DKK1 and activating Wnt/β-catenin signaling pathway. Cancer Biol Ther.

[170] Gao X, Du HJ, Zhang RX, Li C, Wang HG, Xuan Q (2019). Overexpression of cancer susceptibility candidate 2 inhibited progression of hepatocellular carcinoma cells. J Cell Physiol.

